# Unraveling the Expression Patterns of Immune Checkpoints Identifies New Subtypes and Emerging Therapeutic Indicators in Lung Adenocarcinoma

**DOI:** 10.1155/2022/3583985

**Published:** 2022-02-07

**Authors:** Jinguo Zhang, Xinghua Han, Lin Lin, Jian Chen, Feng Wang, Qi Ding, Li Hao, Lingyu Wang, Jie Wei, Yong Wang, Yueyin Pan

**Affiliations:** Department of Medical Oncology, The First Affiliated Hospital of USTC, Division of Life Science and Medicine, University of Science and Technology of China, Hefei, China

## Abstract

Immune checkpoint genes (ICGs) play pivotal roles in tumor immune microenvironment (TIME), and thus, targeting them represents a promising strategy for cancer immunotherapy. However, the genetic landscape of ICGs in lung adenocarcinoma (LUAD) is still unknown. Herein, we comprehensively evaluated the ICG expression profiles of 1439 LUAD samples and linked ICG expression patterns with infiltration of immune cells, clinical features, and response to immune checkpoint blockade (ICB). The ICGscore was developed to quantify ICG expression patterns of individual patient by principal component analysis algorithms. Three distinct ICG expression patterns and three ICG-related genomic clusters were determined, which were implicated in different clinical outcomes, level of immune infiltrates, and biological process. LUAD patients were subdivided into high- and low-ICGscore subgroups. Patients with higher ICGscore were characterized by favorable survival outcomes, increased immune cell infiltration, and enhanced expression of ICGs. Further analysis revealed that lower ICGscore was associated with greater tumor mutation loads and higher mutation rates of *TTN*, *KEAP1*, and *ZFHX4*. High ICGscore has the potential to be a robust indicator in clinical benefit of immunotherapy. Taken together, unraveling the ICG expression patterns will advance our understanding of heterogeneity of TIME and guides more effective immunotherapeutic strategies in LUAD.

## 1. Introduction

Immune checkpoint system, as an important immune inhibitory signaling, participates in maintaining the immune homeostasis in humans and modulating the intensity of the immune response in peripheral tissues, as well as controlling tolerance against self-antigens [1, 2]. Immunotherapy currently in clinical use is aimed at reversing the immunosuppressive tumor environment by various immune checkpoint inhibitors (ICIs) [3]. Beyond these classic immune checkpoint factors, several novel immune checkpoint genes (ICGs) were successively found one after another [4–6]. The biological interplay between tumor cells and the immune system is orchestrated by a complex set of regulatory networks. Immune system is required to maintain the appropriate balance between T cell activation to defend against tumor cells and preventing autoimmunity, which is tightly regulated by an array of cell surface receptors and ligands [7]. To achieve an optimal effector function, T cells require at least T cell receptor (TCR) activation (signal one) and costimulation (signal two). The first signal is mediated by the interaction of TCR with antigen peptides presented by the major histocompatibility complex (MHC) [8]. A second synergistic signal is supported by T cell costimulatory receptor and its ligands on antigen-presenting cells (APCs) such as CD80-CD86, CD40 ligand-CD40, and ICOS-ICOS ligand [9, 10]. Meanwhile, immunoinhibitory receptors and its ligands (e.g., CTLA-4-CD80, PD1-PD-L1) play a pivotal role in inhibiting T cell activation and maintenance of immune tolerance [11]. These immune checkpoints are essential for establishing immune homeostasis, eradicating pathogens efficiently, and protecting organs from unnecessary damage [12]. Therefore, dissecting the genetic landscape of immune checkpoint pathway can facilitate our understanding of its dysregulation in tumor and provide unique opportunities for antitumor immunity.

To survive in immune pressure, various solid tumors including non-small-cell lung cancer (NSCLC) develop several mechanisms to escape from immune surveillance, mainly through expressing immune checkpoint molecules [13]. Lung adenocarcinoma (LUAD), the most common subtype of NSCLC, is characterized by high mutation rate of driver genes including *KRAS*, *EGFR*, *MET*, *BRAF*, *ALK*, and *TP53* [14]. Further, high tumor mutation burden (TMB) and strong immunogenicity were observed in LUAD patients [15]. Currently, the advent of ICIs provides unprecedented opportunities for NSCLC patients with no candidate driver gene mutation or acquired resistance to targeted therapies [16]. PD-1 blockade pembrolizumab has received FDA approval as first-line therapy for advanced NSCLC with >50% tumor cells expressing PD-L1 [17, 18]. However, clinical benefits from ICIs (anti-PD-1/L1 or anti-CTLA4) have been observed in only a minority of patients. Hence, evaluation of novel biomarkers or prediction signatures to define the appropriate subgroups of patients who can clinically benefit from ICIs is warranted. Concomitantly, the efficacy of immunotherapy is greatly influenced by the complex tumor immune microenvironment (TIME) such as infiltrating immune cells, cancer-associated fibroblast, and crosstalks with immune checkpoint pathways [19, 20], which indicates a critical need for a deeper understanding of how immune checkpoint molecules orchestrate the TIME landscape heterogeneity.

In this study, we reported a comprehensive analysis of immune cell infiltration patterns mediated by multiple immune checkpoint molecules through integrating the genomic and gene expression profiles of 1439 LUAD samples based on The Cancer Genome Atlas (TCGA) LUAD dataset and Gene Expression Omnibus (GEO) database. Herein, we delineated the genetic alteration landscape of ICGs and identified three distinct immune checkpoint patterns in LUAD. Additionally, we established a scoring scheme to quantify the immune checkpoint patterns in individual tumors for predicting the prognosis of LUAD patients. Our findings highlighted that the immune checkpoint pathway plays an integral role in modulating individual TME features, molecular typing, and individual management of LUAD.

## 2. Materials and Methods

### 2.1. LUAD Dataset Source and Preprocessing

The public transcriptome expression data and clinical information of LUAD samples were obtained from TCGA (https://cancergenome.nih.gov/) and GEO (https://www.ncbi.nlm.nih.gov/geo/) database. A total of 1439 LUAD patients were collected for analysis, including the TCGA-LUAD dataset (*N* = 535), GSE72094 cohort (*N* = 442) [21], and GSE68465 (*N* = 462) [22]. Patients without complete clinical data were excluded from all analyses. We directly downloaded the normalized matrix expression files for GEO microarray data. As to the TCGA-LUAD dataset, RNA sequencing data (FPKM values) were downloaded from TCGA Genomic Data Commons (GDC, https://portal.gdc.cancer.gov/) and then transformed into transcripts per kilobase million (TPM) values. To eliminate batch effects among different GEO datasets, we performed batch effect correction using the “Sva” R package [23]. The somatic mutation and copy number variation (CNV) data of LUAD were acquired from TCGA database and UCSC Xena database (http://xena.ucsc.edu/), respectively. We plotted the CNV landscape of ICGs in human chromosomes by R package “RCircos.”

### 2.2. Consensus Molecular Clustering of ICGs

Due to the few ICGs annotated by GPL96 platform, we did not include the GSE68465 dataset for clustering analysis. We conducted a systematic literature review and identified 43 prevalent ICGs in LUAD for further analysis (Supplementary Table S1). ICGs are a series of receptors and their ligands that act as active signal, inhibitory signal, or both to induce immune response [24]. We performed unsupervised clustering analysis to identify distinct ICG expression patterns according to 43 ICG expression and stratified LUAD patients into different molecular subtypes for further analysis. The best-fitting number of clusters was determined based on cluster stability, cophenetic, dispersion, and silhouette. The R package “ConsensusClusterPlus” was applied to conduct the consensus clustering with 1000 times repetitions [25].

### 2.3. Gene Set Variation Analysis (GSVA) and Estimation of Immune Cell Infiltration

To explore the biological factors in different ICG patterns, we utilized GSVA enrichment analysis with R package “GSVA.” The gene set of “c2.cp.kegg.v7.2.symbols” was downloaded from the MsigDB online database (http://www.gsea-msigdb.org/gsea/downloads.jsp). The top biological 20 terms were selected with adjusted *P* < 0.05. The single-sample gene-set enrichment analysis (ssGSEA) algorithm was adopted to estimate the abundance of 23 immune cell infiltrations in LUAD TIME. Gene panel for each immune cell type was acquired via referencing the reported literature [26]. The enrichment scores for each sample were calculated and normalized to the range of expression from 0 to 1.

### 2.4. Screening of the Differentially Expressed Genes (DEGs) between Distinct ICG Phenotypes and Functional Enrichment Analysis

To determine ICG-related differentially expressed genes (DEGs) among different ICG clusters, the “limma” method was applied to identify DEGs with the filtering criteria of adjusted *P* < 0.001 [27]. Functional annotation of DEGs by gene ontology (GO) and Kyoto Encyclopedia of Genes and Genomes (KEGG) pathway analyses was performed with R package “clusterProfiler,” “org.Hs.eg.db,” “enrichplot,” and “ggplot2.” The top 30 biological terms were shown with *P* < 0.05.

### 2.5. Generation of the ICG Signature Score

We developed an ICG signature score (ICGscore) to quantify the ICG expression pattern of individual patients with LUAD using principal component analysis (PCA). Briefly, the overlapping DEGs among different ICG clusters were extracted and then, a univariate Cox regression model was employed to perform prognosis analysis for further screening with Cox *P* < 0.01. The genes that were significantly correlated with overall survival (OS) were extracted for further analysis (Supplementary Table S2). PCA was carried out based on the expression profiles of selected gene. Principal component 1 (PC1) and principal component 2 (PC2) were extracted to calculate ICGscore. The formula we defined was similar to previous studies [28, 29]: ICGscore = ∑(PC1*i* + PC2*i*), where *i* represents the expression of ICG-related genes.

### 2.6. Correlation between ICG Signature Score and Immune Cell Infiltration, TMB, and Immunophenoscore (IPS)

The associations of ICGscore with immune cell infiltration were assessed according to the results of ssGSEA using spearman correlation analysis. We calculated TMB scores of each sample based on TCGA LUAD mutation data. Charoentong et al. developed a scoring scheme to quantify tumor immunogenicity termed immunophenoscore (IPS) using machine learning [26]. IPS was generally considered the indicator for ICI response. We collected the immunophenoscores of TCGA LUAD patients from The Cancer Immunome Atlas (TCIA) online tool (https://tcia.at/). Herein, a comparison of IPS between low and high ICGscore was carried out based on CTLA-4 and PD-1 expression status.

### 2.7. Statistical Analysis

All statistical analyses in this study were processed by R-4.0.1. Statistical comparisons between two groups were calculated using the Wilcoxon rank test, while comparisons between three or more groups were performed by Kruskal-Wallis tests. Regarding the survival analysis, we employed the R package “survival” and “survminer” for Kaplan–Meier analysis. The “surv-cutpoint” function was adopted to define the optimal cutoff for dividing samples into high and low subgroups. The CNV landscape of ICGs in human chromosomes was plotted by R package “RCircos.” The waterfall plot was visualized with R package “maftools” to present the mutation profiles in low- and high-ICGscore subtype. All statistical comparisons were two-tailed, with a statistically significant *P* value < 0.05.

## 3. Results

### 3.1. Landscape of Genetic Variation of ICGs in LUAD

**Figure 1:**
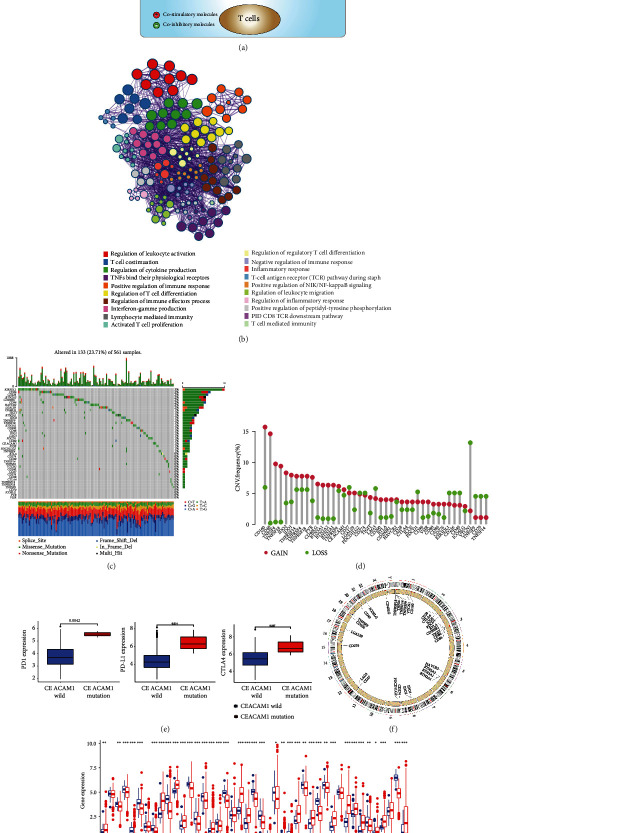
The landscape of genetic and expression alterations of immune checkpoint genes in lung adenocarcinoma. (a) A brief description of the representative immune checkpoint molecules involved in the antitumor immunity. Mechanisms of costimulatory and coinhibitory interactions among tumor cells, T cells, and APC in the tumor microenvironment. (b) Metascape GO analysis of 43 ICGs showed the significant biological process enriched terms. Different colours correspond to indicated enrichment terms. (c) The mutation frequency of 43 ICGs in TCGA-LUAD cohort. Each column represented an individual sample. The number on the right indicated the mutation frequency for each gene. The right histogram summarized the percentage of each variant type. The stacked barplot below indicated fraction of conversions, and the upper barplot showed TMB for an individual sample. (d) The CNV variation frequency of ICGs in TCGA-LUAD cohort. The vertical axis represented the alteration frequency. The deletion frequency, green dot; the amplification frequency, red dot. (e) The effects of CEACAM1 mutation status on PD-L1, PD-1, and CTLA-4 expression. All gene expression values are expressed as TPM. (f) The location of CNV alteration of representative ICGs on chromosomes. (g) The mRNA expression of 43 m6A regulators between normal tissues and lung adenocarcinoma tissues. All gene expression values are expressed as TPM. Normal, blue; tumor, red (^∗^*P* < 0.05; ^∗∗^*P* < 0.01; ^∗∗∗^*P* < 0.001).

In our study, we focused on the roles of 43 representative ICGs in LUAD. In the TME, antitumor immune response is finely tuned by complex costimulatory and coinhibitory signals among tumor cells, T cells, and antigen-presenting cells (APC), which are responsible for driving or dampening the antitumor immune response (Figure 1(a)). We further conducted Metascape GO analyses of 43 ICGs, and networks of significant enrichment biological processes are displayed in Figure 1(b). Among 561 TCGA LUAD samples, 133 cases harbored ICG mutations at a frequency of 23.71%. Our findings showed that *KIR3DL1* exhibited the highest mutation frequency followed by *CD86* and *CD226*, while *VSIR*, *PVR*, *ICOSLG*, and *CD70* did not present any known mutation (Figure 1(c)). CNV alteration analysis revealed that amplifications in copy number of most ICGs were observed. In contrast, a small number of genes (e.g., *PD-L1*, *PD-1*, *LAG3*, *VTCN1*, and *TNFSF9*) had a wide range of CNV deletions (Figure 1(d)). Intriguingly, *CEACAM1* mutation was associated with high expression of PD-L1, PD-1, and CTLA-4 (Figure 1(e)), indicating *CEACAM1* mutation status might serve as a potential biomarker for predicting response to immunotherapy. Additionally, *CD96* mutation substantially decreased the expression of CD96 and CTLA-4 (Supplementary Figure 1). RCircos plot further showed the distribution of CNV alterations of several ICGs on human chromosomes (Figure 1(f)). Further differential expression analysis demonstrated that 37 of 43 ICGs were aberrantly expressed in LUAD at mRNA level (Figure 1(g)). Univariate Cox regression analysis and Kaplan–Meier (KM) curves were employed to investigate the prognostic value of ICGs for LUAD patients. The results of prognosis analysis of ICGs are presented in Supplementary Table S3. The above results depicted the overall genetic variation and expression difference landscape of ICGs, which further supported the prominent role of ICGs in mediating LUAD occurrence and progression.

### 3.2. Identification of Distinct ICG Expression Patterns in LUAD

**Figure 2:**
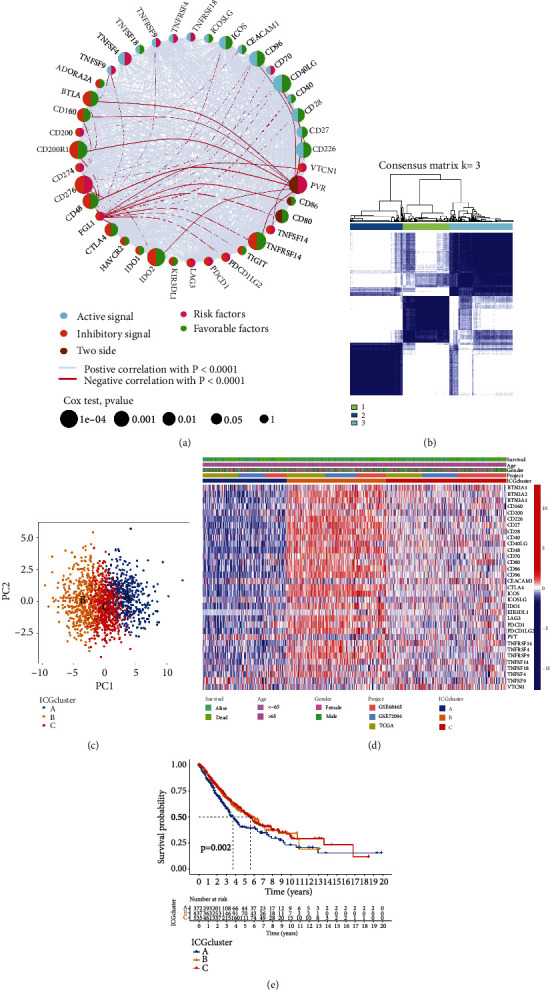
Identification of distinct ICG expression subtypes in LUAD. (a) The interactions of ICGs in LUAD. Active signal, light blue; inhibitory signal, orange; two-side signal, brown. Pink semicircle represented the risk factors of prognosis; green semicircle represented the protective factors of prognosis. The size of the circle represented the effect of ICGs on the prognosis, and the statistical significance was calculated by the Log-rank test. The lines among different ICGs indicated their interactions, and negative correlation was marked with bright red and positive correlation with gray. (b) Consensus clustering matrix for *k* = 3 based on ICG expression. (c) Principal component analysis of ICG expression to distinguish different ICG subtypes. (d) Unsupervised clustering of ICG expression to classify patients into three ICG subtypes. The ICG clusters, datasets, gender, age, and survival status were used as patient annotations. (e) Kaplan-Meier curves of OS for three LUAD subtypes from TCGA-LUAD, GSE72094, and GSE68465. The numbers of patients in ICG-A, ICG-B, and ICG-C subtypes are 372, 437, and 535, respectively.

We combined two GEO datasets (GSE72094 and GSE68465) and TCGA-LUAD cohort to create a final metacohort. The crosstalk involving in host antitumor immune responses between costimulatory and coinhibitory signal was complex. The comprehensive interaction profile of ICG correlations and their prognostic value for LUAD patients were visualized as the checkpoint regulator network (Figure 2(a)). We next performed consensus clustering analysis to classify LUAD samples into different ICG patterns, and three distinct ICG clusters were identified using unsupervised clustering (Figure 2(b) and Supplementary Figure 2). We named these clusters ICGclusters A-C and principal component analysis of ICGcluster showed obvious segregation among three ICG clusters (Figure 2(c)). A striking difference of ICG expression profile was observed between distinct ICG clusters. ICGcluster-A displayed the lowest checkpoint gene expression, while ICGcluster-B exhibited the highest relative abundance of checkpoint genes (Figure 2(d)). Survival analysis further demonstrated that ICGcluster-A had the worst prognosis in the metacohort (Figure 2(e), *P* = 0.002, Log-rank test).

### 3.3. The Distinct ICG Expression Patterns Characterized by Immune Cell Infiltration

**Figure 3:**
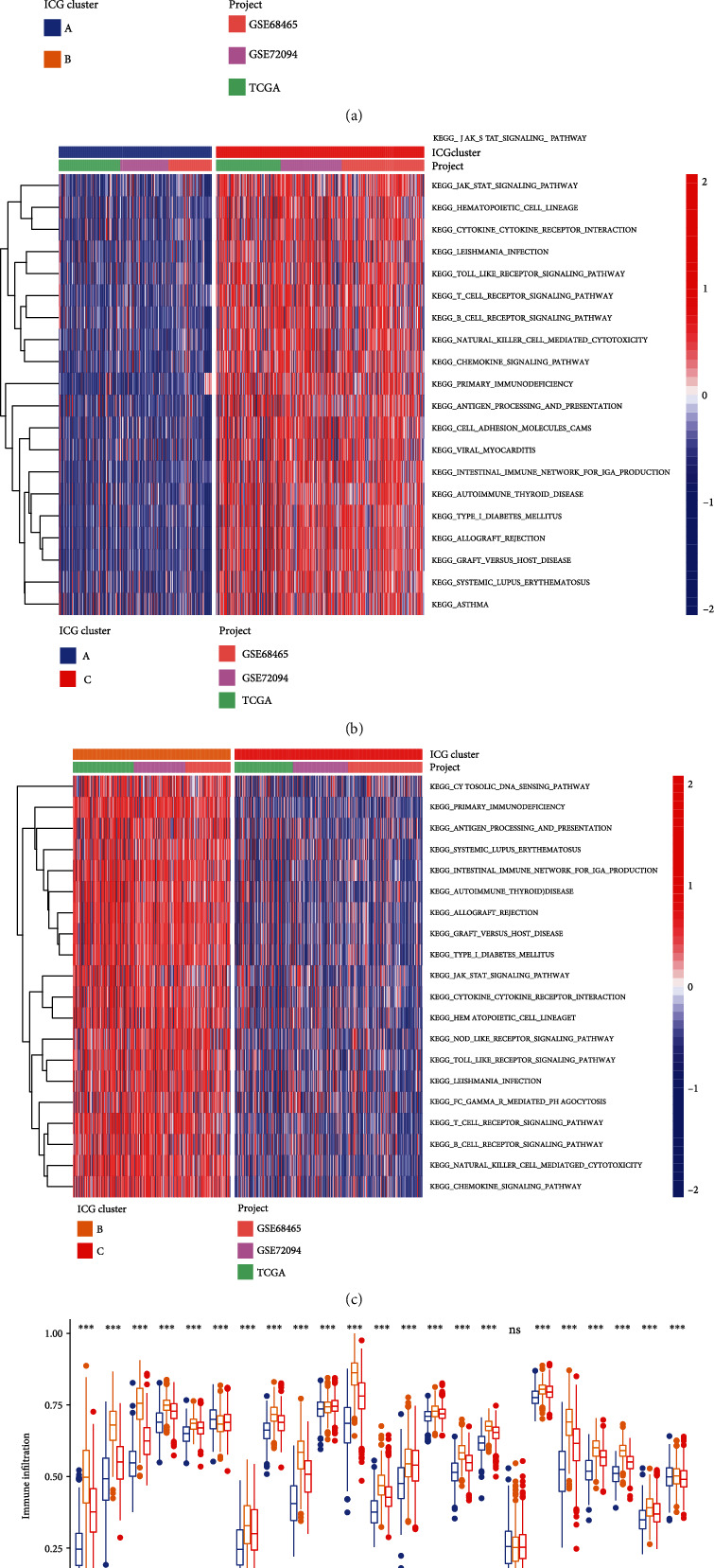
Biological pathways TME characteristics of each ICG subtypes. (a–c) Heat map showed the GSVA score of biological pathways in different ICG subtypes. Red indicated activated pathways, and blue indicated inhibited pathways. (a) ICG-A cluster vs. ICG-B cluster; (b) ICG-A cluster vs. ICG-C cluster; (c) ICG-B cluster vs. ICG-C cluster. (d) The abundance of each TME-infiltrating immune cells in three ICG subtypes. The lines in the boxes indicated the median value. The top and bottom ends of the boxes were interquartile range of values. The statistical significance was calculated by the Kruskal-Wallis *H* test (ns: not significant; ^∗∗^*P* < 0.01; ^∗∗∗^*P* < 0.001).

To investigate the biological process among these distinct ICGs patterns, heat map showed differences in biological pathway activities determined by GSVA between different ICG clusters. As shown in Figure 3(a), compared with ICGcluster-A, ICGcluster-B was markedly enriched in nod-like receptor signaling pathways, toll-like receptor signaling pathways, and adaptive immune response pathways. In addition, ICGcluster-C was highly enriched in JAK-STAT signaling pathway, cytokine receptor interaction, and multiple immune-related signaling compared with ICGcluster-A (Figure 3(b)). Comparisons of the biological behaviors between ICGcluster-B and ICGcluster-C are presented in Figure 3(c). We also noticed that ICGcluster-A was prominently enriched in metabolism-related pathway such as aminoacyl biosynthesis, tricarboxylic acid cycle, and oxidative phosphorylation (Supplementary Table S4). We further calculated ssGSEA scores to quantify the abundances of 23 immune-infiltrating cells in different ICG patterns (Figure 3(d)). A series of antitumor immune cells such as activated CD8 T cell, activated CD4 T cell, activated dendritic cell, and natural killer T cells were mainly enriched in ICGcluster-B subtype, while ICGcluster-A subtype presented relatively low abundances of these immune-infiltrating cells. Based on the results above, we identified ICGcluster-B as a high-immunogenicity LUAD subtype characterized by “hot” immune status and abundant immune cell infiltration.

### 3.4. Generation of Checkpoint Gene Signatures and Functional Annotations

**Figure 4:**
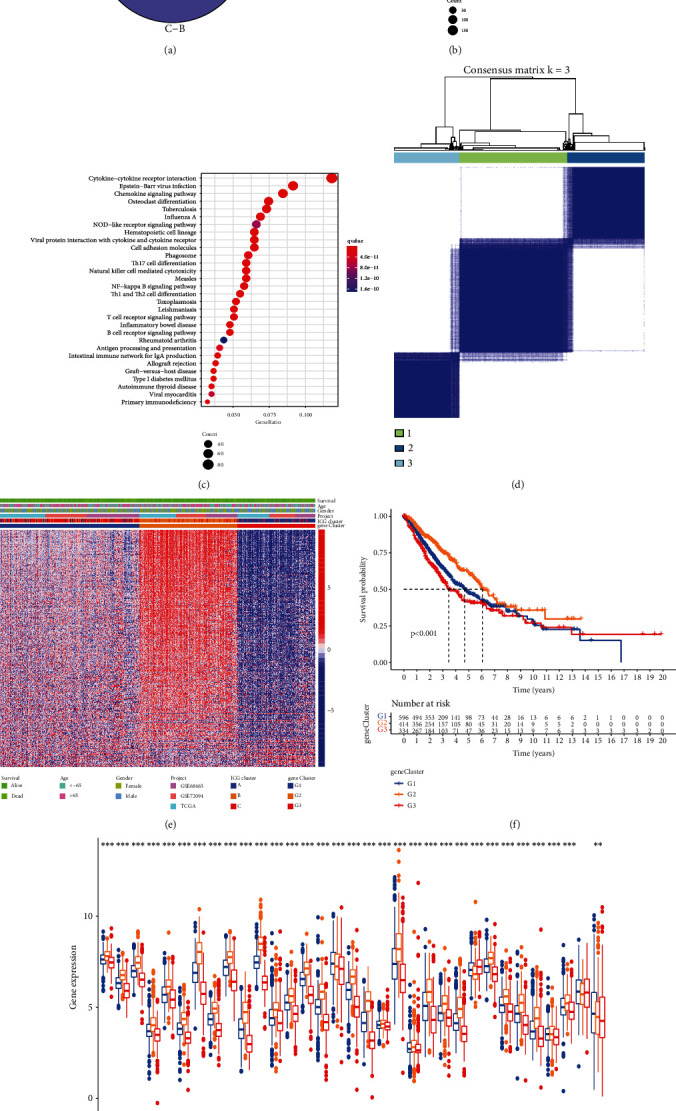
Construction of ICG-related gene signatures and functional annotation. (a) 1173 ICG-related differentially expressed genes (DEGs) between three ICG subtypes were shown in the Venn diagram. (b) Functional enrichment for ICG-related genes using GO annotation analysis. The size of circle represented the number of genes enriched, and *q* value is measured from blue to red. (c) Functional enrichment for ICG-related genes using KEGG pathway analysis. The size of the circle represented the number of genes enriched, and *q* value is measured from blue to red. (d) The identification of ICG-related gene signatures by consensus clustering matrix for *k* = 3. (e) Unsupervised clustering of overlapping ICG-related DEGs to divide patients into different genomic subtypes. The ICG-related gene clusters, ICG clusters, datasets, gender, age, and survival status were used as patient annotations. (f) The survival curves of the ICG-related gene signatures were plotted by the Kaplan-Meier plotter. The numbers of patients in ICG-G1, ICG-G2, and ICG-G3 subtypes are 596, 414, and 334. (g) The expression of ICGs in different ICG-related gene signatures. The statistical significance was calculated by the Kruskal-Wallis *H* test (^∗∗^*P* < 0.01; ^∗∗∗^*P* < 0.001). All gene expression values are expressed as TPM.

To further explore the genetic alterations across three ICG expression patterns, we determined the potential ICG-related DEGs using “limma” R package. In total, 1173 overlapping DEGs were recognized as ICG-related signature and results were illustrated in a Venn diagram (Figure 4(a)). GO analysis revealed that these genes were predominantly enriched in “T cell activation,” “external side of plasma membrane,” and “cytokine receptor binding” (Figure 4(b)). KEGG pathway enrichment analysis further demonstrated that DEGs were mainly involved in immune-related pathways such as cytokine−cytokine receptor interaction, chemokine signaling pathway, and T cell receptor signaling pathway (Figure 4(c)). After screening DEGs by the univariate Cox regression model, we further performed consensus clustering analysis to produce a robust genomic subtype of LUAD patients. Unsupervised consensus clustering also suggested three ICG-related genomic clusters, and we defined it as ICG-G1, ICG-G2, and ICG-G3 (Figure 4(d) and Supplementary Figure 3). We noted that ICG-G2 patients were largely overlapped with ICGcluster-B subtype, and patients with alive status were mainly focused on the ICG-G2 subtype (Figure 4(e)). Further survival analysis indicated that ICG-G2 was correlated with better prognosis, while ICG-G3 was correlated with worse OS (Figure 4(f)). Beyond this, ICG-G2 patients exhibited higher expression of most checkpoint genes than other subtypes (Figure 4(g)), which agreed with the previous results of ICG pattern profiles.

### 3.5. Construction of the ICG Signature Score and Exploration of Its Clinical Significance

**Figure 5:**
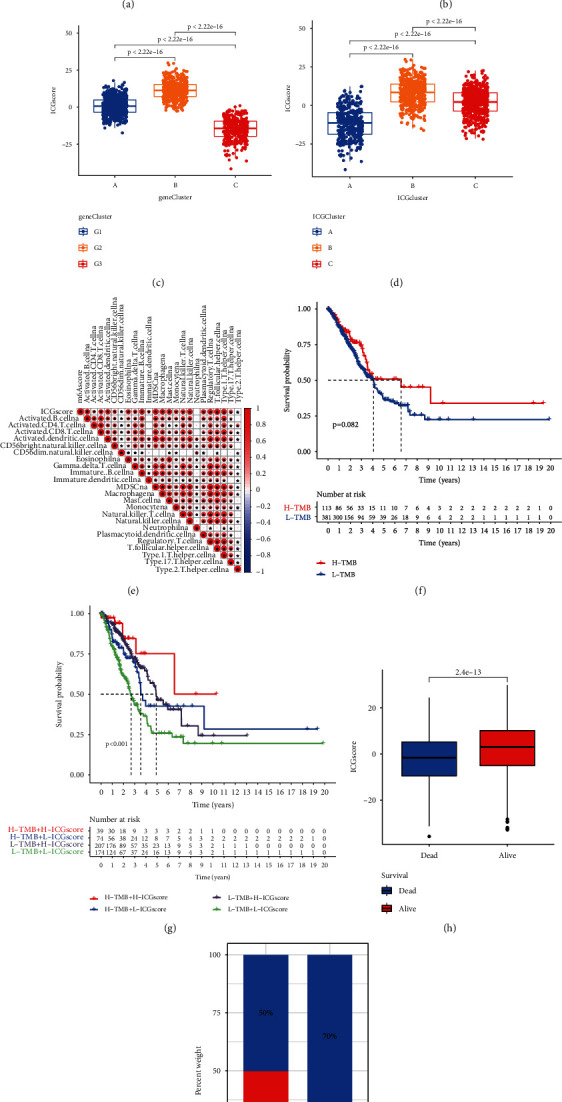
Construction of ICGscore and exploration of its clinical significance. (a) Kaplan-Meier curves for high- and low-ICGscore patient groups in our metacohort. Log-rank test, *P* < 0.001. (b) Alluvial diagram of ICG clusters in groups with different ICG-related gene signatures, m6Sig score, and survival status. (c) The correlation between ICGscore and ICG-related gene signatures. (d) The correlation between ICGscore and ICG clusters. (e) Heat map showed the relationship between ICG score and immune cell infiltration. (f) Kaplan-Meier curves for high- and low-TMB patient groups in TCGA-LUAD dataset. Log-rank test, *P* = 0.082. (g) Survival analyses for subgroup patients stratified by both ICGscore and TMB using Kaplan-Meier curves. (h) The effect of ICGscore on survival status in LUAD. (i) The proportion of survival status in the high- and low-ICGscore group.

**Figure 6:**
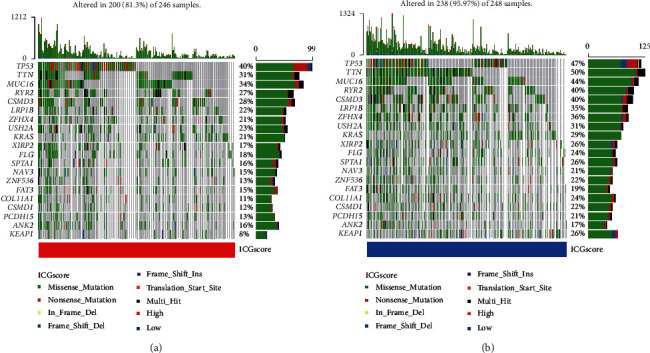
Relative distribution of tumor mutation load in the high- and low-ICGscore group. The waterfall plot of tumor somatic mutation established by patients with high ICGscore (a) and low ICGscore (b). Each column represented an individual sample. The upper barplot showed TMB for an individual sample. The number on the right indicated the mutation frequency for each gene. The right histogram summarized the percentage of each variant type.

To quantify the ICG TME pattern of individual LUAD sample, we developed a scoring system termed as ICGscore. Notably, high ICG signature score was associated with favorable survival outcomes of LUAD patients (Figure 5(a)). The characteristics of individual patients were illustrated by the alluvial diagram (Figure 5(b)). These results demonstrated that ICG-G2 with the ICGcluster-B subtype was linked to a higher ICGscore, while ICG-G3 with ICGcluster-A subtype had a lower ICGscore. ICGcluster-C subtype had a high overlap with ICG-G1, while this overlapping population exhibited low ICGscore with a favorable prognosis. We also noticed that ICGcluster-B and ICG-G2 subtype showed the highest ICG signature score (Figures 5(c) and 5(d)). To illustrate the relationship between ICGscore and immune cell infiltration, a heat map of the correlation analysis indicated that ICG signature score was markedly positively related to the infiltration of activated B cells, CD4 T cells, CD8 T cells, Type1 T helper cells, and other antitumor immune cells (Figure 5(e)). Since TMB levels correlate with immunotherapeutic efficacy in some types of tumors, we further explored the prognostic value of TMB level combined with our ICG signature score in LUAD. In TCGA-LUAD cohort, a high level of TMB tended to predict a longer survival time, but the difference was not statistically significant (*P* = 0.082, Figure 5(f)). However, we were surprised to observe that high TMB level and high ICG score were closely related to a favorable survival, whereas LUAD patients with low TMB level and low ICG score had the worst prognosis (Figure 5(g)). The significant prognosis benefits were further validated in our metacohort LUAD patients with high ICGscore compared to those with low ICGscore (Figures 5(h) and 5(i)). Then, we investigated the distribution differences of significant somatic mutation in different ICGscore groups of TCGA-LUAD cohort with “maftools” package. The mutation landscapes of the waterfall plot revealed that the low-ICGscore group presented a higher mutation burden than the high-ICGscore group, with the percentage of the top 20 most significant mutated gene 95.97% versus 81.3% (Figures 6(a) and 6(b)). Of these, *TTN* (50% vs. 31%), *KEAP1* (26% vs. 8%), and *ZFHX4* (36% vs. 21%) had higher mutation rates in the low-ICGscore group.

### 3.6. The Predictive Value of ICGscore for the Immunotherapeutic Benefits

**Figure 7:**
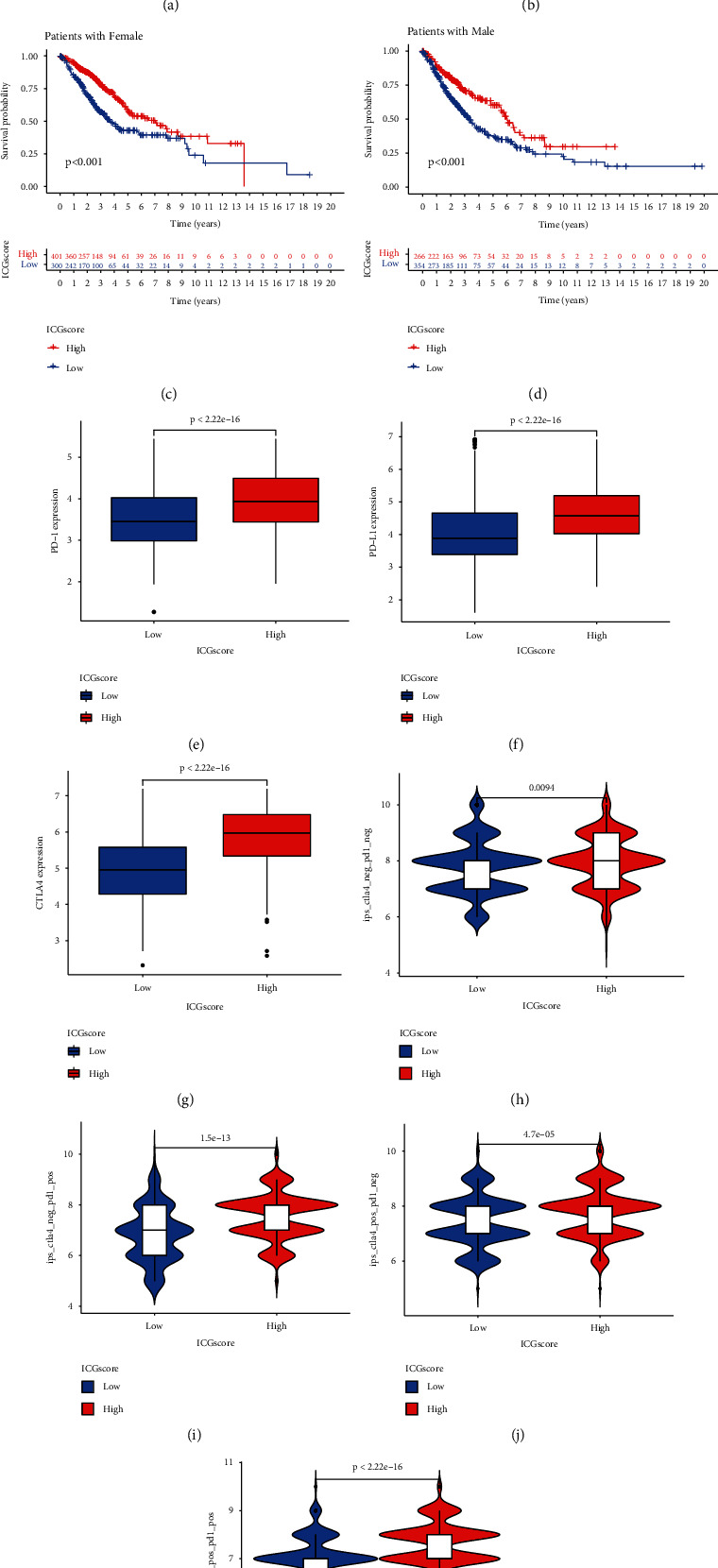
The predictive value of ICGscore in LUAD. (a–d) The ICGscore retained its prognostic value in multiple subgroups of LUAD patients. Survival analysis in the low- and high-ICGscore group adjusted by age and gender. (e–g) The expression of immune checkpoint members including PD-1, PD-L1, and CTLA-4 in high- and low-ICGscore LUAD groups. All gene expression values are expressed as TPM. (h–k) The relative distribution of IPS adjusted by CTLA-4 or PD-1 expression was compared between ICGscore high versus low groups in TCGA-LUAD dataset.

To evaluate the stability and applicability of our ICGscore, we further analyzed the effect of patient characteristics on the prognosis of ICGscore. We stratified LUAD samples by age and gender and found that ICGscore signature still had a satisfactory prediction of prognosis (Figures 7(a)–7(d)). Immune checkpoint inhibitors represented by antibodies against PD-1, PD-L1, and CTLA-4 constitute the current frontier in cancer therapy. We wondered whether the ICGscore could serve as a predictive signature for immune checkpoint blockade therapy. We first noted that an increased level of PD-1, PD-L1, and CTLA4 expression was found in the high-ICGscore subgroup (Figures 7(e)–7(g)), which implied a potential response to immune checkpoint blockade therapy. Further analysis using IPS scheme demonstrated that high ICGscore had the potential to be a robust indicator for immunotherapies in TCGA-LUAD cohort, especially for patients with PD-1 or CTLA-4 positive expression (Figures 7(h)–7(k)). These findings strongly indicated that ICGscore significantly correlated to antitumor immune and immunotherapy response in LUAD.

## 4. Discussion

The LUAD is characterized by high degree of heterogeneity in terms of its clinical behavior and molecular landscapes [30]. Although great strides are being made in ICI therapy for LUAD, only a small number of patients respond to ICIs [31]. Intense efforts have been made to develop precise predictive immunooncology biomarkers, such as TMB, PD-L1 expression, and tumor-infiltrating immune cells [32]. Nevertheless, to date, there are still no unified standards or single indicator to select LUAD patients from receiving ICIs. Cancer immunotherapy is usually achieved by targeting ICGs such as antibody inhibition of PD-1 and CTLA-4, but its clinical efficacy largely depends on the individual tumor immune microenvironment and immune-related regulatory networks [33], which underscores the importance of understanding how immune checkpoint genes orchestrate TME patterns and affect patients' prognostic outcomes. Unraveling the role of ICG-mediated TME patterns can help to provide important implications for rational immunotherapy strategies. Recently, several studies have revealed the immune signature for prediction of ICI efficacy in LUAD. Yi et al. conducted a 17-gene immune signature to predict survival and response to ICI treatment of LUAD [15]. The study of Guo et al. reported a 10 immune-related gene model that could facilitate the management of immunotherapy in LUAD [34]. In addition, Zhou et al. also established a nine-gene signature based on immune checkpoints to aid prognostic analysis in LUAD [35]. The aforementioned studies were confined to several-gene model, while the antitumor immune response effect was influenced by complex components of TIME. Here, we first investigated the landscape of genetic variation of ICGs in LUAD and identified LUAD subtype based on ICG expression patterns and ICG-related genomic profiles. We also developed a scoring scheme ICGscore based on ICG-related DEG expression using PCA rather than based on selected immune-related genes. In this study, 43 prevalent ICGs were identified based on a comprehensive literature search. Metascape GO analyses further confirmed that ICGs indeed played a sizable role in T cell costimulation, positive or negative regulation of immune response. We then portrayed the landscape of ICG genomic alterations in TCGA-LUAD cohort. Overall, genomic alterations of ICGs presented a relatively low proportion of LUAD patients. Previous studies reported the independent prognosticator role of CEACAM1 in lung adenocarcinoma [36, 37]. Here, we showed that *CEACAM1* mutation status was found to correlate with the expression of PD-L1, PD-1, and CTLA1, underlining the potential predictive value of CEACAM1 in ICI therapy. We next revealed three distinct ICG patterns which were correlated with different patients' prognosis and immune phenotypes. ICGcluster-B was characterized by abundant immune cell infiltration in TME with elevated ICG expression levels, indicating the potential benefits of immunotherapy. Previous literature reported that baseline levels of tumor-infiltrating CD8+ T lymphocytes cells, and B cells were correlated with the better prognosis of LUAD [38, 39]. In contrast, the ICGcluster-A subgroup with poor prognosis was markedly enriched in metabolism-related pathway, supporting the essential role of metabolic flexibility in mediating disease progression. Based on the above findings, we proposed that LUAD patients with the ICGcluster-B expression pattern might most benefit from the treatment of ICIs.

We further compared the mRNA transcriptome differences among different ICG-related patterns and found that DEGs mainly participated in immune relevant phenotypes and cytokine interactions. Three ICG-related genomic clusters were further identified that were strongly associated with survival time. Similar to the ICGcluster-B pattern, the ICG-G2 subgroup achieved better survival outcome and higher expression of ICGs than those with other ICG-related genomic clusters. Given the individual heterogeneity of immune checkpoint pathways, we thereby designed a quantification system named the ICGscore to evaluate different ICG patterns for individual patients. As a result, the ICGcluster-B and ICG-G2 subtype exhibited a higher ICGscore, suggesting ICGscore was a positive prognostic indicator in LUAD. This might be partly explained by abundant infiltration of antitumor immune cells in the high-ICGscore subgroup. High somatic mutation burden represents a uniform feature of patients who had a better response to ICIs [40]. Furthermore, the relationships between TMB and response rates after PD-1 inhibitors treatments have been demonstrated in a pancancer study [41]. Here, we combined our ICGscore and TMB to predict the prognosis of LUAD and found that LUAD patients with high level of TMB and ICGscore had the longest OS. These findings confirmed our hypothesis that the ICG pathway patterns could provide guidance for the clinical practice.

Identification of the cancer driver genes is also strongly guiding therapeutic strategies with the development of targeted therapies. In this study, we noticed that the mutation rates of *TTN*, *KEAP1*, and *ZFHX4* were obviously increased in the low-ICGscore group. It has been reported that *TTN*/*ZFHX4* missense triggered the development of tumor-associated antigens and could predict a good prognosis in lung cancer [42, 43]. *KEAP1* mutation has a dramatic effect on the tumor immune microenvironment of LUAD and may also serve as a predictive biomarker for immunotherapy [44]. A previous study suggested that *KEAP1* mutations were correlated with shorter survival and reduced efficacy of immunotherapy of LUAD [45]. Further analysis revealed that the high-ICGscore subgroup exhibited an increased expression of ICGs, which confirmed that our ICGscore was markedly associated with the antitumor immune activity. Finally, we also verified the predictive value of the ICGscore using IPS immune response predictor. Potential limitations of our study should be acknowledged. First, since the included studies were of retrospective datasets in our work, therefore, further prospective cohort of LUAD patients receiving ICIs is warranted to validate our findings. Second, we reviewed the literature and chose the representative ICGs in LUAD; however, some new identified ICGs need to be incorporated into ICG expression patterns for future investigation. Thirdly, in this study, we only tested the accuracy of the ICGscore in LUAD. We thus hope that ICI-based immunotherapy in pancancer could further support our conclusions. Moreover, some LUAD patients with high ICGscore exhibited a poor survival outcome; thus, more clinicopathological parameters might require integration into our prediction models to improve accuracy.

## 5. Conclusions

In conclusion, we comprehensively evaluated 43 ICG expression profile among 1439 LUAD patients and systematically linked ICG-related patterns with immune cell-infiltrating characteristics and immunotherapeutic efficacy. In clinical practice, we identified three distinct ICG clusters and ICG-related genomic clusters and developed the ICGscore to evaluate the characterization of TME cell infiltration for individual patients. Our ICGscore was able to assess the abundances of tumor-infiltrating immune cells, tumor mutation burden, patients' survival outcomes, and the potential immunotherapeutic benefits. More significantly, this integrated analysis added another new insight into the molecular subtype of LUAD and provided a novel concept for guiding ICI immunotherapy. Our findings also advanced the understanding of the complex interplay between immune checkpoint pathways and tumor immune microenvironment.

## Data Availability

Publicly available datasets or databases were analyzed in the present study. This data can be found here: TCGA database (https://portal.gdc.cancer.gov) and GEO database (https://www.ncbi.nlm.nih.gov/geo/), under the accession numbers GSE72094 and GSE68465.

## Abbreviations

ICGs: Immune checkpoint genes
TIME: Tumor immune microenvironment
LUAD: Lung adenocarcinoma
ICB: Immune checkpoint blockade
ICIs: Immune checkpoint inhibitors
TCR: T cell receptor
MHC: Major histocompatibility complex
APC: Antigen-presenting cells
NSCLC: Non-small-cell lung cancer
TMB: Tumor mutation burden
GEO: Gene Expression Omnibus
TCGA: The Cancer Genome Atlas
GSVA: Gene Set Variation Analysis
CNV: Copy number variation
DEGs: Differentially expressed genes
GO: Gene ontology
KEGG: Kyoto Encyclopedia of Genes and Genomes
ICGscore: ICG signature score
OS: Overall survival.

## Supplementary Materials

Supplementary Materials Supplementary Figure 1: the effects of *CD96* mutation status on CD96 and CTLA-4 expression. Supplementary Figure 2: cumulative distribution function curve and relative change of delta area for identification of ICG expression patterns. Supplementary Figure 3: cumulative distribution function curve and relative change of delta area for identification of ICG-related signatures. Supplementary Table S1: the overview of selected 43 representative immune checkpoint genes in LUAD. Supplementary Table S2: the results of Cox regression analysis for overlapping differentially expressed genes. Supplementary Table S3: univariate Cox regression and Kaplan–Meier (KM) analysis of ICGs in LUAD patients. Supplementary Table S4: the top20 biological pathways involving in ICGcluster-A subtype compared with other subtypes.
