# Supplementary material for: Unraveling the Expression Patterns of Immune Checkpoints Identifies New Subtypes and Emerging Therapeutic Indicators in Lung Adenocarcinoma
Source: Oxid Med Cell Longev. 2022 Feb 7;2022:3583985. doi: 10.1155/2022/3583985 (PMC8843963; doi:10.1155/2022/3583985)
Supplement: Supplementary Materials — Supplementary Figure 1: the effects of CD96 mutation status on CD96 and CTLA-4 expression. Supplementary Figure 2: cumulative distribution function curve and relative change of delta area for identification of ICG expression patterns. Supplementary Figure 3: cumulative distribution function curve and relative change of delta area for identification of ICG-related signatures. Supplementary Table S1: the overview of selected 43 representative immune checkpoint genes in LUAD. Supplementary Table S2: the results of Cox regression analysis for overlapping differentially expressed genes. Supplementary Table S3: univariate Cox regression and Kaplan–Meier (KM) analysis of ICGs in LUAD patients. Supplementary Table S4: the top20 biological pathways involving in ICGcluster-A subtype compared with other subtypes. [file 3583985.f1.zip › 3583985.f1/Supplementary Table S1.pdf]

Table S1: The Overview of selected 43 immune checkpoints genes in LUAD.

Symbol	Type	Type of co-stimulation signal
BTLA	Receptor	Inhibitory signal
BTN2A1	Ligand	TwoSide
BTN2A2	Ligand	Inhibitory signal
BTN3A1	Ligand	Active signal
CD160	Ligand	Inhibitory signal
CD200	Ligand	Inhibitory signal
CD200R1	Ligand	Inhibitory signal
CD226	Receptor	Active signal
CD27	Receptor	Active signal
CD274	Ligand	TwoSide
CD276	Ligand	Inhibitory signal
CD28	Receptor	Active signal
CD40	Receptor	Active signal
CD40LG	Ligand	Active signal
CD48	Ligand	Inhibitory signal
CD70	Ligand	Active signal
CD80	Ligand	TwoSide
CD86	Ligand	TwoSide
CD96	Receptor	Active signal
CEACAM1	Ligand&Receptor	Active signal
CTLA4	Receptor	Inhibitory signal
HAVCR2	Receptor	Inhibitory signal
ICOS	Receptor	Active signal
ICOSLG	Ligand	Active signal
IDO1	Ligand	Inhibitory signal
IDO2	Ligand	Inhibitory signal
KIR3DL1	Receptor	Inhibitory signal
LAG3	Receptor	Inhibitory signal
LGALS9	Ligand	Inhibitory signal
PDCD1	Receptor	Inhibitory signal
PDCD1LG2	Ligand	TwoSide
PVR	Ligand	TwoSide
TIGIT	Receptor	Inhibitory signal
TNFRSF14	Ligand	TwoSide
TNFRSF18	Receptor	Active signal
TNFRSF4	Receptor	Active signal
TNFRSF9	Receptor	Active signal
TNFSF14	Ligand	Inhibitory signal
TNFSF18	Ligand	Active signal
TNFSF4	Ligand	Active signal
TNFSF9	Ligand	Active signal
VSIR	Ligand	Inhibitory signal
VTCN1	Ligand	Inhibitory signal
