# Supplementary material for: Unraveling the Expression Patterns of Immune Checkpoints Identifies New Subtypes and Emerging Therapeutic Indicators in Lung Adenocarcinoma
Source: Oxid Med Cell Longev. 2022 Feb 7;2022:3583985. doi: 10.1155/2022/3583985 (PMC8843963; doi:10.1155/2022/3583985)
Supplement: Supplementary Materials — Supplementary Figure 1: the effects of CD96 mutation status on CD96 and CTLA-4 expression. Supplementary Figure 2: cumulative distribution function curve and relative change of delta area for identification of ICG expression patterns. Supplementary Figure 3: cumulative distribution function curve and relative change of delta area for identification of ICG-related signatures. Supplementary Table S1: the overview of selected 43 representative immune checkpoint genes in LUAD. Supplementary Table S2: the results of Cox regression analysis for overlapping differentially expressed genes. Supplementary Table S3: univariate Cox regression and Kaplan–Meier (KM) analysis of ICGs in LUAD patients. Supplementary Table S4: the top20 biological pathways involving in ICGcluster-A subtype compared with other subtypes. [file 3583985.f1.zip › 3583985.f1/Supplementary Table S2.pdf]

Table S2: The results of Cox regression analysis for overlaping DEGs

Gene Name	HR	HR.95L	HR.95H	pvalue
CD2	0.828163	0.754169	0.909417	7.87E-05
SLAMF1	0.765784	0.681884	0.860007	6.57E-06
PTPRC	0.868657	0.799643	0.943627	0.000857
IL10RA	0.852492	0.770118	0.943676	0.002083
CD48	0.833293	0.763778	0.909135	4.07E-05
TRAF3IP3	0.746565	0.664284	0.839037	9.31E-07
CD247	0.852787	0.775118	0.938238	0.001081
CD53	0.860702	0.781068	0.948456	0.002459
CD6	0.753272	0.674021	0.84184	5.87E-07
CXCR6	0.871372	0.786391	0.965537	0.008543
GZMK	0.913862	0.855398	0.97632	0.007576
PTPN7	0.849569	0.768613	0.939053	0.001419
IL2RG	0.887184	0.812108	0.969199	0.007967
ITK	0.851292	0.775582	0.934393	0.000704
ARHGAP25	0.792304	0.709568	0.884686	3.51E-05
CD3E	0.861421	0.786131	0.943922	0.00139
ICOS	0.874777	0.80891	0.946007	0.000809
WAS	0.828887	0.740604	0.927694	0.00109
SASH3	0.792682	0.717247	0.876051	5.28E-06
MAP4K1	0.790012	0.711874	0.876726	9.17E-06
GIMAP4	0.861605	0.781593	0.949807	0.00274
NCKAP1L	0.849913	0.768273	0.940228	0.001599
CD3D	0.861791	0.792964	0.936592	0.000461
LCK	0.861782	0.785761	0.945157	0.001594
IL16	0.71812	0.634911	0.812234	1.37E-07
BIN2	0.872163	0.80615	0.943581	0.000659
CORO1A	0.790999	0.720686	0.868172	7.96E-07
ACAP1	0.817108	0.743329	0.898209	2.87E-05
EVI2B	0.85978	0.785309	0.941312	0.001082
SIT1	0.86374	0.791113	0.943034	0.00108
LPXN	0.800867	0.706336	0.908049	0.00053
IRF8	0.860676	0.782023	0.94724	0.002151
ITGAL	0.862839	0.791329	0.940812	0.000831
CD3G	0.863533	0.799266	0.932969	0.000201
GVINP1	0.813857	0.728212	0.909573	0.000283
CD37	0.83541	0.766966	0.909962	3.73E-05
CD27	0.870748	0.807669	0.938754	0.00031
IKZF1	0.816994	0.737992	0.904454	9.81E-05
DOCK2	0.863911	0.788622	0.946388	0.001664
KLRB1	0.798066	0.728881	0.873818	1.09E-06
SELL	0.875885	0.807025	0.950619	0.001513
GPR65	0.852654	0.773593	0.939796	0.001324
PSTPIP1	0.778434	0.694728	0.872227	1.59E-05
TRAT1	0.905021	0.846192	0.96794	0.003612
GMFG	0.83331	0.753719	0.921305	0.00037
CYTH4	0.841009	0.74389	0.950807	0.00568
CTLA4	0.885311	0.814158	0.962682	0.004377
CD96	0.879322	0.804666	0.960906	0.004499
CCR2	0.744585	0.665362	0.833241	2.77E-07
CCR7	0.797144	0.731722	0.868416	2.11E-07
CD4	0.842782	0.762652	0.931331	0.000792
HCLS1	0.853815	0.773849	0.942045	0.001633
HLA-E	0.811722	0.711297	0.926326	0.001964
BTK	0.814526	0.738798	0.898017	3.78E-05
IL7R	0.835709	0.773155	0.903325	6.15E-06
GIMAP6	0.869914	0.797734	0.948626	0.001614

CCL19	0.900161	0.855047	0.947656	6.09E-05
P2RY10	0.854525	0.7874	0.927373	0.000166
SELPLG	0.863662	0.782215	0.953589	0.003728
PPP1R16B	0.895259	0.82888	0.966953	0.004879
LCP1	0.866563	0.786661	0.95458	0.003711
GFI1	0.877227	0.799489	0.962524	0.005662
TAP1	1.158515	1.051628	1.276268	0.00289
ARHGEF6	0.760424	0.679742	0.850684	1.70E-06
MYO1F	0.801898	0.712464	0.902559	0.000253
HLA-DMB	0.850308	0.785479	0.920488	6.13E-05
PRKCB	0.785767	0.700742	0.881108	3.69E-05
CXCL11	1.087018	1.027361	1.150139	0.003764
CD180	0.832804	0.752257	0.921976	0.000423
LY9	0.809513	0.729498	0.898305	6.90E-05
UBASH3A	0.832514	0.758337	0.913947	0.000118
SAMHD1	0.872696	0.788573	0.965794	0.008465
CLEC10A	0.823173	0.753465	0.899331	1.63E-05
ZAP70	0.842894	0.767032	0.926259	0.000382
PIK3CG	0.859595	0.779756	0.94761	0.002351
CD226	0.843005	0.74346	0.955879	0.007727
NCF4	0.870539	0.785234	0.965112	0.008418
P2RY13	0.863874	0.809789	0.92157	9.16E-06
CXCR3	0.859798	0.785254	0.941419	0.001096
CD79B	0.869392	0.806346	0.937366	0.000268
CD52	0.850395	0.790982	0.914271	1.16E-05
INPP5D	0.819592	0.723837	0.928013	0.001698
GPR18	0.858618	0.788415	0.935072	0.000461
HLA-DOB	0.868412	0.806031	0.935621	0.000208
LTB	0.83699	0.777038	0.901568	2.70E-06
ICAM3	0.718541	0.636417	0.811263	9.41E-08
ITGB2	0.877644	0.809069	0.95203	0.001665
LST1	0.839993	0.767834	0.918932	0.000142
DOCK10	0.830999	0.757105	0.912106	9.77E-05
LSP1	0.87687	0.803279	0.957202	0.003304
FLI1	0.801629	0.707299	0.90854	0.000537
HLA-DPB1	0.846306	0.787568	0.909424	5.44E-06
RHOH	0.842075	0.765485	0.926328	0.000411
FNBP1	0.810478	0.703535	0.933678	0.003609
PIK3CD	0.721246	0.630562	0.824973	1.87E-06
GPR171	0.888387	0.827271	0.954017	0.001136
PLEKHO1	0.865239	0.780621	0.959028	0.005839
HLA-DRA	0.865898	0.807328	0.928717	5.59E-05
TRIM22	0.818713	0.741747	0.903666	7.16E-05
MNDA	0.903783	0.844026	0.967771	0.003748
CD19	0.83803	0.768429	0.913935	6.49E-05
BTN2A2	0.702421	0.604126	0.81671	4.38E-06
HLA-DPA1	0.872261	0.810495	0.938735	0.000265
LTA	0.847049	0.749543	0.957239	0.007806
CD79A	0.903007	0.851096	0.958085	0.000732
MS4A1	0.870462	0.823993	0.919552	7.19E-07
STAP1	0.844858	0.781488	0.913366	2.26E-05
CD74	0.832153	0.762648	0.907993	3.65E-05
CD69	0.885367	0.819475	0.956558	0.002032
CD5	0.848282	0.773837	0.929889	0.000446
IGSF6	0.847195	0.764466	0.938877	0.001562
SEMA4D	0.809303	0.69843	0.937777	0.004885
TLR7	0.891656	0.826955	0.961419	0.002848
RASSF2	0.842525	0.762992	0.930348	0.000706

PLCG2	0.794888	0.692344	0.912619	0.001124
IRF4	0.843678	0.772612	0.92128	0.000153
CYLD	0.780457	0.663196	0.918451	0.002845
FGD2	0.847448	0.747871	0.960284	0.009448
CD28	0.780989	0.688577	0.885805	0.000119
HLA-DOA	0.86523	0.787702	0.950389	0.002508
IGHM	0.921725	0.873887	0.972182	0.002722
CIITA	0.851576	0.766575	0.946003	0.002748
LY86	0.856132	0.782585	0.93659	0.0007
CD40LG	0.776121	0.711696	0.846377	9.91E-09
SPI1	0.874283	0.801687	0.953453	0.002384
CD80	0.819021	0.725141	0.925055	0.001308
PIM2	0.887711	0.812552	0.969822	0.008318
GPSM3	0.781113	0.698841	0.87307	1.36E-05
TNFSF8	0.84565	0.753852	0.948627	0.004243
CARD8	0.740675	0.62196	0.882051	0.000757
HLA-DMA	0.801989	0.737005	0.872703	3.08E-07
BCL11B	0.826212	0.742321	0.919585	0.000475
SPIB	0.865847	0.801243	0.935659	0.000272
VAV1	0.838518	0.75131	0.935848	0.001671
TAP2	1.20271	1.057817	1.367449	0.00483
PTGDS	0.850744	0.798447	0.906466	5.92E-07
SPOCK2	0.850066	0.765849	0.943543	0.002275
CD244	0.840099	0.756555	0.932869	0.001113
APBB1IP	0.865542	0.785225	0.954074	0.003659
RASGRP2	0.693232	0.609351	0.788659	2.58E-08
ITGA4	0.828504	0.747595	0.91817	0.000333
IKZF3	0.866074	0.791155	0.948087	0.001841
GYPC	0.84833	0.763096	0.943085	0.00233
PTPN6	0.725002	0.608232	0.86419	0.000332
NELL2	0.854272	0.77941	0.936324	0.000763
TRANK1	0.795716	0.696743	0.908747	0.000746
CD1D	0.813999	0.737791	0.89808	4.07E-05
TNFRSF17	0.930069	0.884643	0.977828	0.004546
KLRG1	0.822244	0.713973	0.946935	0.006591
LY75	0.861575	0.781073	0.950374	0.002911
C11orf21	0.756625	0.649508	0.881407	0.000343
LGALS2	0.901739	0.84351	0.963988	0.002391
DENND1C	0.725711	0.629512	0.836611	9.93E-06
PTK2B	0.766039	0.671353	0.87408	7.52E-05
MFNG	0.800753	0.694045	0.923868	0.002326
N4BP2L1	0.711131	0.620531	0.814958	9.45E-07
DEF6	0.831875	0.739574	0.935695	0.002158
FCRL2	0.878735	0.808757	0.954768	0.002264
BIRC3	1.101508	1.024629	1.184156	0.008817
CD22	0.747153	0.669246	0.83413	2.13E-07
SKAP1	0.867724	0.791748	0.95099	0.002407
EVL	0.835861	0.732024	0.954426	0.008069
CCR6	0.811549	0.73841	0.891933	1.47E-05
PECAM1	0.820906	0.727092	0.926825	0.001436
GAS7	0.858977	0.768153	0.96054	0.007675
GRAP2	0.733196	0.631777	0.850895	4.39E-05
SYK	0.857393	0.768055	0.957124	0.006134
TNFRSF14	0.761629	0.671081	0.864394	2.48E-05
LPAR6	0.845887	0.756559	0.945761	0.00329
ITM2A	0.887778	0.8131	0.969315	0.007928
ARHGDIB	0.739714	0.662889	0.825443	7.09E-08
CD33	0.861753	0.779005	0.953292	0.003869

DPEP2	0.832584	0.759425	0.91279	9.44E-05
P2RY14	0.803716	0.732525	0.881825	3.88E-06
TSPAN32	0.731973	0.639737	0.837506	5.61E-06
CEACAM21	0.765863	0.681309	0.860911	7.86E-06
LAT	0.767989	0.650439	0.906783	0.001843
TM6SF1	0.82634	0.73887	0.924165	0.000833
CHST11	1.236185	1.114886	1.370682	5.73E-05
MAP3K14	0.842246	0.743744	0.953794	0.006821
CCDC69	0.878645	0.802875	0.961566	0.004927
FYN	0.782125	0.698294	0.876019	2.15E-05
ACP5	0.863864	0.784977	0.95068	0.002743
FCN1	0.830911	0.760582	0.907744	4.04E-05
MAN2B1	0.770734	0.663309	0.895558	0.000673
RASGRP1	0.878397	0.804035	0.959637	0.004068
SOCS1	0.819696	0.723357	0.928865	0.001829
CD83	0.813067	0.735301	0.899058	5.47E-05
ENPP2	0.855738	0.77671	0.942806	0.001626
LILRA4	0.880672	0.804029	0.96462	0.006231
EAF2	0.860521	0.773588	0.957223	0.0057
KCNAB2	0.826355	0.73078	0.93443	0.002355
FLT3	0.796081	0.703271	0.901139	0.000311
KCTD12	0.843954	0.76739	0.928158	0.000472
TDO2	1.124393	1.031505	1.225645	0.007697
PVRIG	0.83289	0.741315	0.935777	0.002092
LMO2	0.81742	0.721972	0.925487	0.001461
P2RX1	0.821934	0.733999	0.920405	0.000682
HERPUD1	0.749124	0.649711	0.863748	7.00E-05
NLRP1	0.770387	0.693826	0.855397	1.04E-06
PIK3IP1	0.712092	0.624971	0.811357	3.40E-07
KAT2B	0.760024	0.673273	0.857952	9.10E-06
PARP12	1.317221	1.126609	1.540081	0.000551
FCHSD2	0.715264	0.611158	0.837104	2.97E-05
BANK1	0.841505	0.77952	0.908418	9.85E-06
NAAA	0.81147	0.702087	0.937894	0.004685
CHIT1	0.926093	0.884982	0.969114	0.000919
ABCA6	0.842092	0.761772	0.930881	0.000778
SLC2A5	1.221202	1.105037	1.349578	8.91E-05
CAMK4	0.752027	0.633784	0.89233	0.001094
TCN2	0.83541	0.742086	0.94047	0.002925
PLSCR1	1.223651	1.080749	1.385447	0.001445
APOL2	1.208512	1.065862	1.370254	0.003124
TNFRSF13B	0.842429	0.757613	0.936741	0.00154
S1PR4	0.852969	0.760492	0.956692	0.006605
NMI	1.373926	1.194058	1.580888	9.11E-06
CYSLTR1	0.788374	0.716474	0.867489	1.10E-06
VOPP1	1.291078	1.127365	1.478565	0.000222
ARRB2	0.754881	0.65827	0.865673	5.71E-05
CFP	0.834693	0.736514	0.945959	0.004653
GIT2	0.819292	0.710943	0.944155	0.005888
SAMD9	1.13106	1.036799	1.233891	0.005538
MICAL1	0.804146	0.715259	0.90408	0.000265
DCN	0.901771	0.834955	0.973935	0.008479
MEF2C	0.77581	0.695456	0.865448	5.36E-06
BCL2	0.772015	0.680753	0.875513	5.55E-05
PLCB2	0.750977	0.671051	0.840423	6.10E-07
GP1BA	0.792992	0.693611	0.906611	0.000686
ST6GAL1	0.832004	0.750004	0.922969	0.000512
CD101	0.840192	0.749261	0.94216	0.002888

APOL1	1.205912	1.11246	1.307214	5.38E-06
LYL1	0.8189	0.741674	0.904167	7.71E-05
IFNAR2	0.792433	0.683238	0.919079	0.002102
IGHD	0.933159	0.893461	0.974621	0.001815
MYCBP2	0.775881	0.669144	0.899644	0.000778
KCNA3	0.867433	0.803745	0.936167	0.000257
ST8SIA1	0.879895	0.80746	0.958827	0.003509
CADM3	0.855798	0.76341	0.959368	0.007548
TLR6	1.246549	1.079313	1.439697	0.002714
PLA1A	0.906567	0.847011	0.97031	0.004665
PML	1.320148	1.135543	1.534763	0.000302
ICAM2	0.852884	0.757914	0.959753	0.008242
STK17B	0.821958	0.717939	0.941047	0.004509
NFATC1	0.781979	0.672572	0.909184	0.001384
ARPC2	1.365538	1.079584	1.727235	0.009358
MMP12	1.062594	1.017648	1.109526	0.0059
RALGDS	0.756509	0.635582	0.900445	0.001689
RCBTB2	0.798111	0.704902	0.903645	0.000372
ZNF671	0.799071	0.711916	0.896896	0.000141
MAP3K3	0.642633	0.545456	0.757123	1.25E-07
ELMO1	0.802285	0.725902	0.886706	1.59E-05
EFNA3	1.185162	1.091505	1.286855	5.24E-05
ADPRH	0.831437	0.728422	0.949021	0.006233
DOCK4	0.816485	0.719659	0.926337	0.001644
GNG7	0.72133	0.641236	0.811427	5.34E-08
NDST2	0.684364	0.541139	0.865497	0.001547
IL2	0.801428	0.718336	0.894132	7.38E-05
CLIC2	0.873635	0.814776	0.936746	0.000147
DNASE2	0.70228	0.575649	0.856766	0.000494
PTPRO	0.821918	0.72211	0.935521	0.002988
OAS3	1.243248	1.124821	1.374142	2.02E-05
IARS2	1.297276	1.103432	1.525173	0.001622
CEACAM4	0.865518	0.78198	0.957981	0.005289
TXNDC15	0.678827	0.568184	0.811015	1.98E-05
NMB	1.243117	1.135663	1.360737	2.38E-06
ATG7	1.344217	1.100728	1.641567	0.003717
MYBPC2	0.845179	0.778918	0.917077	5.39E-05
MAST3	0.704555	0.612335	0.810665	9.96E-07
PAOX	0.739762	0.61966	0.883143	0.000854
RENBP	0.87757	0.804336	0.957472	0.003309
CBFA2T3	0.746688	0.660226	0.844473	3.28E-06
CR2	0.88624	0.83907	0.936062	1.51E-05
ARSB	1.263271	1.05827	1.507983	0.009686
NCK1	1.273089	1.093129	1.482676	0.001902
DOK1	0.69261	0.579387	0.827958	5.51E-05
COL3A1	1.123593	1.038629	1.215508	0.003676
RAB40B	0.796401	0.705451	0.899077	0.000234
ANKRD55	0.762362	0.628689	0.924458	0.005806
ANK2	0.773821	0.682907	0.876837	5.79E-05
SYNRG	0.760255	0.621873	0.92943	0.007498
ATXN1	0.757496	0.657899	0.87217	0.000113
CD1B	0.861257	0.800864	0.926203	5.66E-05
TUBB6	1.164205	1.049778	1.291104	0.003974
S100A8	1.093966	1.037743	1.153234	0.000849
SKAP2	1.275552	1.112216	1.462875	0.000499
TNFRSF25	0.838519	0.748408	0.939479	0.002396
CLC	0.89929	0.829681	0.974739	0.009811
TREML2	0.837098	0.73397	0.954717	0.00803

DLG5	1.225885	1.078663	1.393202	0.001809
BZW2	1.287654	1.120575	1.479645	0.000363
IL27RA	0.873063	0.792876	0.961359	0.005751
VNN1	1.126039	1.065683	1.189813	2.41E-05
GTF2IRD1	1.237496	1.053657	1.453411	0.009406
CCDC88C	0.778386	0.690968	0.876863	3.76E-05
ADAMTS2	1.155595	1.035356	1.289797	0.009886
KRT18	1.276862	1.124367	1.45004	0.000166
TYK2	0.787448	0.661774	0.936989	0.007068
UCP2	0.807494	0.71761	0.908636	0.000383
NINJ1	0.762314	0.660653	0.879619	0.000202
MAFK	1.196604	1.105953	1.294686	7.99E-06
PEX13	1.245986	1.069245	1.451942	0.004836
FAM189B	1.278124	1.121952	1.456035	0.000224
FURIN	1.18828	1.088553	1.297144	0.000115
HERC1	0.78387	0.665789	0.922894	0.003464
OAS1	1.19889	1.10262	1.303564	2.16E-05
PTK6	1.10178	1.028507	1.180272	0.005771
ACSL3	1.276247	1.109129	1.468546	0.000658
CTSH	0.759525	0.70298	0.820618	3.20E-12
PRSS8	0.856804	0.790359	0.928835	0.000175
SNRPF	1.240893	1.080363	1.425275	0.002261
NEIL3	1.255581	1.16825	1.349441	6.10E-10
RECQL4	1.197167	1.108703	1.29269	4.34E-06
TLR5	0.848619	0.756508	0.951945	0.005109
PBK	1.248663	1.169602	1.333069	2.85E-11
FMO4	0.795461	0.701888	0.90151	0.000339
HNRNPC	1.703718	1.299688	2.233349	0.000114
EXOSC2	1.548585	1.298365	1.847026	1.15E-06
TPI1	1.52514	1.315117	1.768704	2.36E-08
GAL	1.122017	1.05265	1.195956	0.000406
PPAT	1.297949	1.147023	1.468735	3.55E-05
SCNN1B	0.847831	0.79994	0.898588	2.63E-08
KNTC1	1.211968	1.097977	1.337793	0.000136
DTX4	0.867296	0.790263	0.951839	0.002699
SLC15A2	0.823882	0.758695	0.89467	4.10E-06
SNRPB	1.331695	1.160667	1.527925	4.42E-05

HR:Hazard ratios; HR.95L:95% lower confidence intervals; HR.95H:95% higher confidence intervals;
