# Supplementary material for: Unraveling the Expression Patterns of Immune Checkpoints Identifies New Subtypes and Emerging Therapeutic Indicators in Lung Adenocarcinoma
Source: Oxid Med Cell Longev. 2022 Feb 7;2022:3583985. doi: 10.1155/2022/3583985 (PMC8843963; doi:10.1155/2022/3583985)
Supplement: Supplementary Materials — Supplementary Figure 1: the effects of CD96 mutation status on CD96 and CTLA-4 expression. Supplementary Figure 2: cumulative distribution function curve and relative change of delta area for identification of ICG expression patterns. Supplementary Figure 3: cumulative distribution function curve and relative change of delta area for identification of ICG-related signatures. Supplementary Table S1: the overview of selected 43 representative immune checkpoint genes in LUAD. Supplementary Table S2: the results of Cox regression analysis for overlapping differentially expressed genes. Supplementary Table S3: univariate Cox regression and Kaplan–Meier (KM) analysis of ICGs in LUAD patients. Supplementary Table S4: the top20 biological pathways involving in ICGcluster-A subtype compared with other subtypes. [file 3583985.f1.zip › 3583985.f1/Supplementary Table S3.pdf]

Table S3. Univariate Cox regression and Kaplan–Meier (KM) analysis of ICG

Gene Name	HR	HR.95L	HR.95H	pvalue	KM-Pvalue
BTLA	0.750127	0.638738	0.880942	0.000456	8.43E-05
BTN2A1	0.744956	0.597431	0.928908	0.008925	0.000742385
BTN2A2	0.642922	0.538438	0.76768	1.05E-06	1.58E-06
BTN3A1	0.869837	0.73324	1.031881	0.10962	0.02902391
CD160	0.705093	0.569989	0.872222	0.001284	3.98E-05
CD200	1.096518	0.94328	1.27465	0.230263	0.062641536
CD200R1	0.666154	0.557301	0.796268	8.10E-06	1.06E-07
CD226	0.704186	0.586495	0.845493	0.000171	2.12E-05
CD27	0.908657	0.829435	0.995446	0.039586	0.01800908
CD274	1.049554	0.946537	1.163783	0.358844	0.001630432
CD276	1.518751	1.250658	1.844311	2.47E-05	2.85E-05
CD28	0.804681	0.696186	0.930085	0.003273	0.000989537
CD40	0.911355	0.810187	1.025157	0.122077	0.034094125
CD40LG	0.720731	0.639172	0.812697	9.05E-08	1.95E-07
CD48	0.825574	0.744544	0.915422	0.000276	7.65E-05
CD70	1.137229	0.98499	1.312997	0.07948	0.006874776
CD80	0.81607	0.712503	0.934691	0.003332	0.002180796
CD86	0.896015	0.793436	1.011857	0.076734	0.043437505
CD96	0.814362	0.729119	0.90957	0.000272	0.000356521
CEACAM1	0.937423	0.847677	1.03667	0.208187	0.011454026
CTLA4	0.849478	0.758924	0.950838	0.004561	0.001115366
HAVCR2	0.935272	0.8325	1.050732	0.259859	0.096756878
ICOS	0.823602	0.729712	0.929572	0.001675	0.001593443
ICOSLG	0.918722	0.648246	1.302054	0.633746	0.122854861
IDO1	0.971638	0.903365	1.04507	0.438919	0.003456773
IDO2	0.689257	0.572224	0.830226	8.87E-05	1.93E-06
KIR3DL1	0.788115	0.566442	1.096538	0.157639	0.076062682
LAG3	1.006437	0.900839	1.124414	0.909668	0.126335261
PDCD1	1.008691	0.874549	1.163408	0.905392	0.09728868
PDCD1LG2	1.006158	0.897303	1.128218	0.916309	0.037748516
PVR	1.50771	1.282106	1.773013	6.88E-07	1.68E-08
TIGIT	0.906218	0.801164	1.025049	0.117248	0.011586379
TNFRSF14	0.72259	0.618405	0.844327	4.31E-05	3.51E-05
TNFRSF18	1.076439	0.962743	1.203561	0.195905	0.053060557
TNFRSF4	1.003707	0.852977	1.181072	0.964454	0.148398104
TNFRSF9	1.014049	0.903695	1.137879	0.812398	0.218462664
TNFSF14	1.062771	0.897858	1.257975	0.479176	0.018798589
TNFSF18	0.827696	0.665994	1.02866	0.088161	0.009946714
TNFSF4	1.201961	1.05058	1.375155	0.007397	0.000104427
TNFSF9	1.056671	0.948147	1.177616	0.318783	0.070161883
VTCN1	1.017056	0.951073	1.087618	0.621183	0.208267787

HR:Hazard ratios; HR.95L:95% lower confidence intervals; HR.95H:95% higher confidence intervals; KM-Pvalue, Pvalue calculated by Kaplan–Meier (KM) analysis

s in LUAD patients.
