# Supplementary material for: Unraveling the Expression Patterns of Immune Checkpoints Identifies New Subtypes and Emerging Therapeutic Indicators in Lung Adenocarcinoma
Source: Oxid Med Cell Longev. 2022 Feb 7;2022:3583985. doi: 10.1155/2022/3583985 (PMC8843963; doi:10.1155/2022/3583985)
Supplement: Supplementary Materials — Supplementary Figure 1: the effects of CD96 mutation status on CD96 and CTLA-4 expression. Supplementary Figure 2: cumulative distribution function curve and relative change of delta area for identification of ICG expression patterns. Supplementary Figure 3: cumulative distribution function curve and relative change of delta area for identification of ICG-related signatures. Supplementary Table S1: the overview of selected 43 representative immune checkpoint genes in LUAD. Supplementary Table S2: the results of Cox regression analysis for overlapping differentially expressed genes. Supplementary Table S3: univariate Cox regression and Kaplan–Meier (KM) analysis of ICGs in LUAD patients. Supplementary Table S4: the top20 biological pathways involving in ICGcluster-A subtype compared with other subtypes. [file 3583985.f1.zip › 3583985.f1/Supplementary Table S4.pdf]

Table S4. The top20 biological pathways involving in ICGcluster-A subtype compared

ICGcluster-A versus ICGcluster-C

id	logFC	AveExpr	P.Value
KEGG_AMINOACYL_TRNA_BIOSYNTHESIS	-0.268161	0.031079	3.82E-33
KEGG_RNA_POLYMERASE	-0.240501	0.021477	2.32E-34
KEGG_ONE_CARBON_POOL_BY_FOLATE	-0.21478	0.0053	6.98E-29
KEGG_CITRATE_CYCLE_TCA_CYCLE	-0.20573	0.025771	6.53E-26
KEGG_DNA_REPLICATION	-0.202052	-0.054898	1.34E-14
KEGG_MISMATCH_REPAIR	-0.19725	-0.02818	5.41E-18
KEGG_BASE_EXCISION_REPAIR	-0.195341	-0.016094	8.56E-24
KEGG_RIBOSOME	-0.195298	-0.001879	3.90E-14
KEGG_RNA_DEGRADATION	-0.194992	0.003206	2.04E-32
KEGG_NON_HOMOLOGOUS_END_JOINING	-0.187692	0.009167	4.90E-19
KEGG_GLYOXYLATE_AND_DICARBOXYLATE_METABOLI	-0.186894	0.002386	1.95E-21
KEGG_SPLICEOSOME	-0.177815	-9.58E-05	1.43E-23
KEGG_PYRIMIDINE_METABOLISM	-0.177812	0.00103	9.66E-34
KEGG_PARKINSONS_DISEASE	-0.176953	0.006112	3.78E-21
KEGG_OXIDATIVE_PHOSPHORYLATION	-0.170902	0.016911	4.85E-20
KEGG_PYRUVATE_METABOLISM	-0.167493	0.0248	4.18E-26
KEGG_TERPENOID_BACKBONE_BIOSYNTHESIS	-0.166903	0.052196	4.05E-14
KEGG_HOMOLOGOUS_RECOMBINATION	-0.163947	-0.034059	1.85E-14
KEGG_NUCLEOTIDE_EXCISION_REPAIR	-0.157432	-0.013158	3.21E-19
KEGG_BASAL_TRANSCRIPTION_FACTORS	-0.156132	-0.002262	2.08E-22

ICGcluster-A versus ICGcluster-B

id	logFC	AveExpr	P.Value
KEGG_AMINOACYL_TRNA_BIOSYNTHESIS	-0.314496	0.017931	3.15E-41
KEGG_TERPENOID_BACKBONE_BIOSYNTHESIS	-0.301349	-0.012263	9.59E-38
KEGG_GLYCOSYLPHOSPHATIDYLINOSITOL_GPI_ANCHO	-0.295665	-0.032057	1.73E-56
KEGG_PROPANOATE_METABOLISM	-0.270413	-0.019421	2.39E-41
KEGG_RNA_POLYMERASE	-0.266906	0.017758	1.30E-36
KEGG_CITRATE_CYCLE_TCA_CYCLE	-0.251441	0.010268	9.14E-34
KEGG_VALINE_LEUCINE_AND_ISOLEUCINE_DEGRADAT	-0.236282	-0.032706	1.96E-33
KEGG_BUTANOATE_METABOLISM	-0.234387	-0.022625	8.81E-42
KEGG_PYRUVATE_METABOLISM	-0.213954	0.007251	1.75E-38
KEGG_OXIDATIVE_PHOSPHORYLATION	-0.206552	0.00527	2.84E-25
KEGG_PHENYLALANINE_METABOLISM	-0.198187	-0.016499	3.81E-23
KEGG_ONE_CARBON_POOL_BY_FOLATE	-0.196994	0.024023	1.34E-23
KEGG_HUNTINGTONS_DISEASE	-0.195415	0.001889	3.26E-40
KEGG_PARKINSONS_DISEASE	-0.183336	0.010327	1.54E-20
KEGG_ASCORBATE_AND_ALDARATE_METABOLISM	-0.179465	0.00473	6.99E-17
KEGG_RNA_DEGRADATION	-0.172533	0.023567	2.07E-23
KEGG_FATTY_ACID_METABOLISM	-0.170122	-0.033208	2.42E-19
KEGG_LYSINE_DEGRADATION	-0.16807	-0.009322	1.69E-33
KEGG_GLYOXYLATE_AND_DICARBOXYLATE_METABOLI	-0.168012	0.020493	2.14E-16
KEGG_SPLICEOSOME	-0.164852	0.014466	4.46E-19
