# Supplementary figures and images for: Unraveling the Expression Patterns of Immune Checkpoints Identifies New Subtypes and Emerging Therapeutic Indicators in Lung Adenocarcinoma
Source: Oxid Med Cell Longev. 2022 Feb 7;2022:3583985. doi: 10.1155/2022/3583985 (PMC8843963; doi:10.1155/2022/3583985)

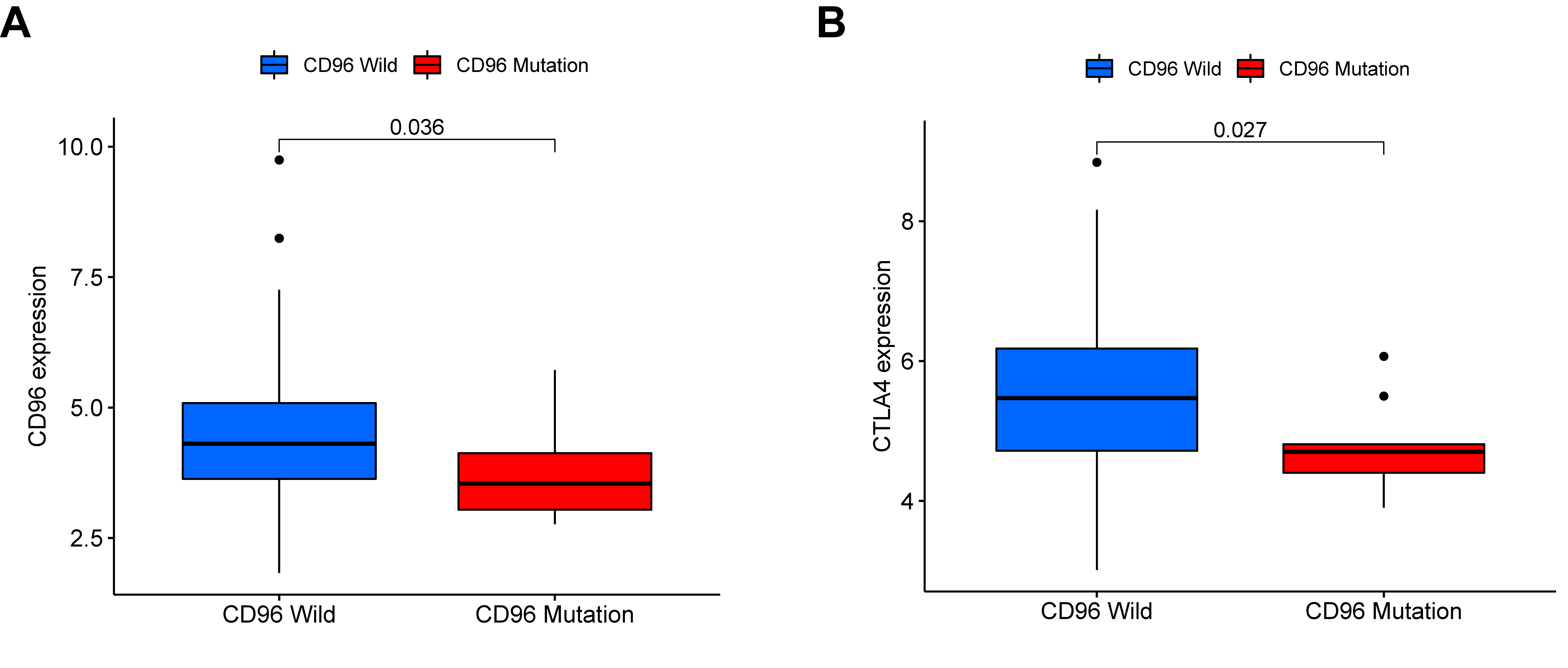

Supplement: Supplementary Materials — Supplementary Figure 1: the effects of CD96 mutation status on CD96 and CTLA-4 expression. Supplementary Figure 2: cumulative distribution function curve and relative change of delta area for identification of ICG expression patterns. Supplementary Figure 3: cumulative distribution function curve and relative change of delta area for identification of ICG-related signatures. Supplementary Table S1: the overview of selected 43 representative immune checkpoint genes in LUAD. Supplementary Table S2: the results of Cox regression analysis for overlapping differentially expressed genes. Supplementary Table S3: univariate Cox regression and Kaplan–Meier (KM) analysis of ICGs in LUAD patients. Supplementary Table S4: the top20 biological pathways involving in ICGcluster-A subtype compared with other subtypes. [file 3583985.f1.zip › 3583985.f1/Supplementary Figure 1.JPG]

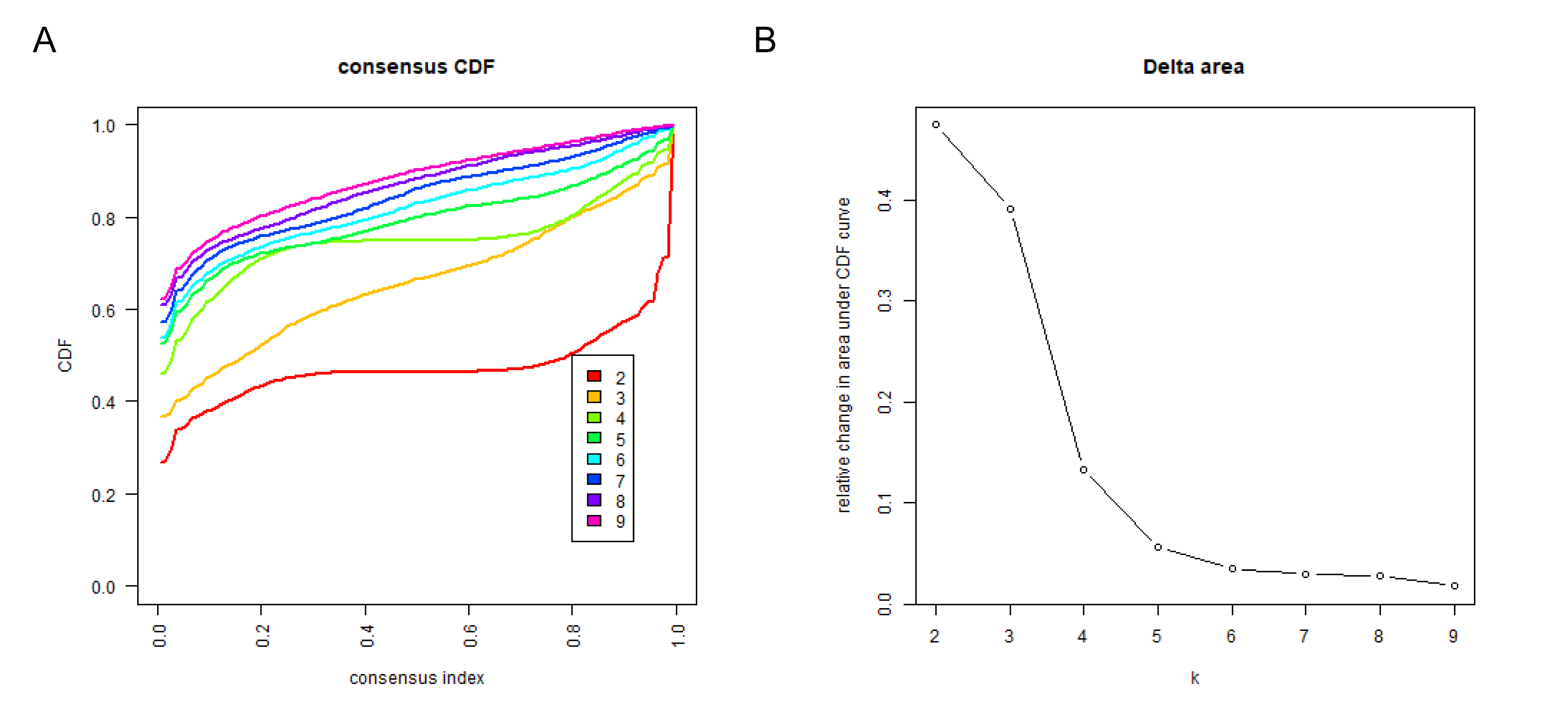

Supplement: Supplementary Materials — Supplementary Figure 1: the effects of CD96 mutation status on CD96 and CTLA-4 expression. Supplementary Figure 2: cumulative distribution function curve and relative change of delta area for identification of ICG expression patterns. Supplementary Figure 3: cumulative distribution function curve and relative change of delta area for identification of ICG-related signatures. Supplementary Table S1: the overview of selected 43 representative immune checkpoint genes in LUAD. Supplementary Table S2: the results of Cox regression analysis for overlapping differentially expressed genes. Supplementary Table S3: univariate Cox regression and Kaplan–Meier (KM) analysis of ICGs in LUAD patients. Supplementary Table S4: the top20 biological pathways involving in ICGcluster-A subtype compared with other subtypes. [file 3583985.f1.zip › 3583985.f1/Supplementary Figure 2.JPG]

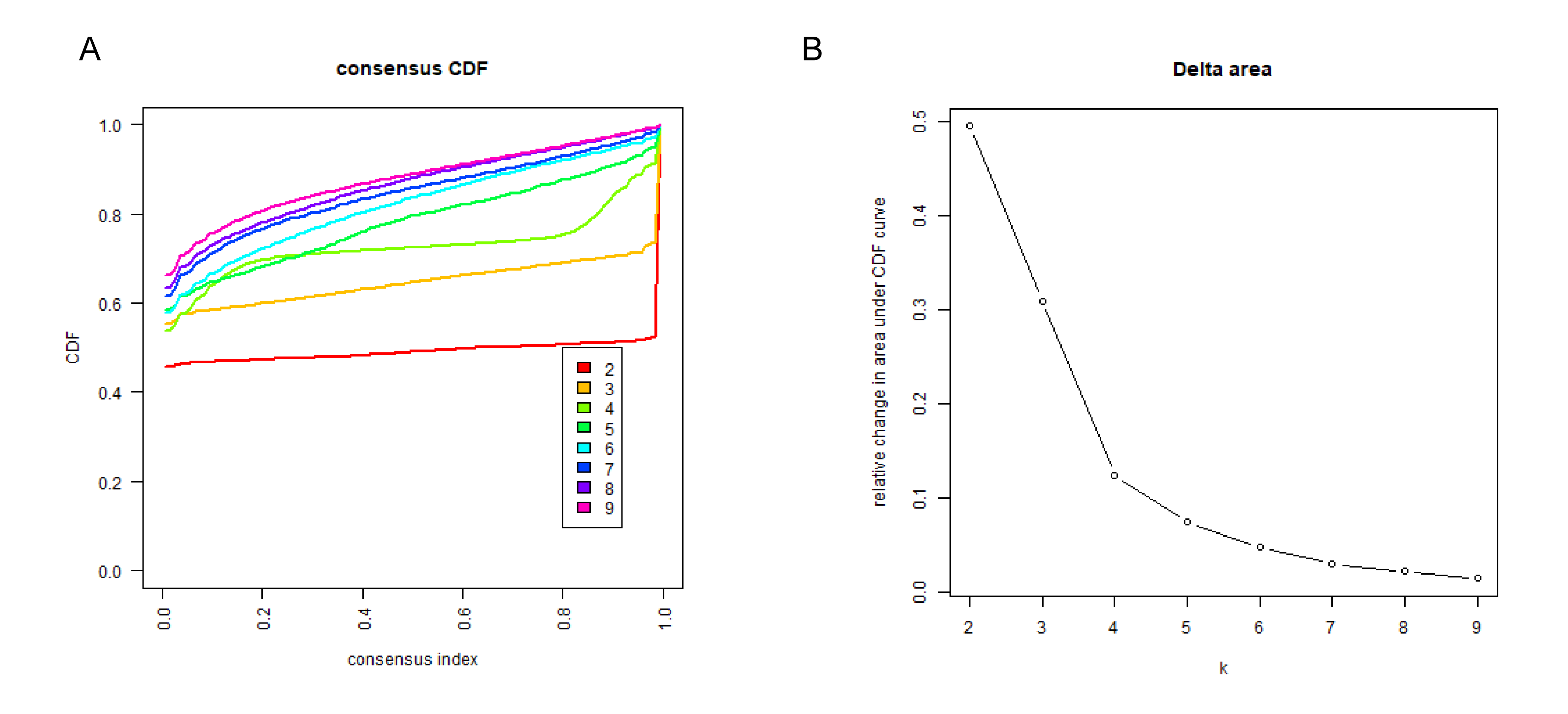

Supplement: Supplementary Materials — Supplementary Figure 1: the effects of CD96 mutation status on CD96 and CTLA-4 expression. Supplementary Figure 2: cumulative distribution function curve and relative change of delta area for identification of ICG expression patterns. Supplementary Figure 3: cumulative distribution function curve and relative change of delta area for identification of ICG-related signatures. Supplementary Table S1: the overview of selected 43 representative immune checkpoint genes in LUAD. Supplementary Table S2: the results of Cox regression analysis for overlapping differentially expressed genes. Supplementary Table S3: univariate Cox regression and Kaplan–Meier (KM) analysis of ICGs in LUAD patients. Supplementary Table S4: the top20 biological pathways involving in ICGcluster-A subtype compared with other subtypes. [file 3583985.f1.zip › 3583985.f1/Supplementary Figure 3.JPG]
